# Functional Brain Activity within the Medial and Lateral Portion of BA10 during a Prospective Memory Task

**DOI:** 10.3233/BEN-2012-129012

**Published:** 2013

**Authors:** Francesco Barban, Giovanni Augusto Carlesimo, Emiliano Macaluso, Carlo Caltagirone, Alberto Costa

**Affiliations:** ^1^Clinical and Behavioural Neurology LaboratoryIRCCS Fondazione Santa LuciaRomeItaly; ^2^Neuroimaging LaboratoryIRCCS Fondazione Santa LuciaRomeItaly; ^3^Institute of NeurologyUniversity of Rome “Tor Vergata”RomeItaly

**Keywords:** Prospective memory, BA10, Gateway hypothesis

## Abstract

In this study we tested the gateway hypothesis of Broadmann area 10 (BA10). With a functional magnetic resonance imaging (fMRI) protocol we manipulated the saliency–stimulus-oriented (SO) attending– and the memory load – stimulus-independent (SI) attending–during a prospective memory (PM) task. We found a significant main effect of the SO manipulation within the medial BA10 and a significant interaction between SI attending and PM task within the left lateral BA10. Our results give experimental support to the gateway hypothesis.

## 1. Introduction

The Brodmann Area 10 (BA10) has been recently found to be critically involved in the integration of the outcome of different cognitive operations for the achievement of goals in complex situations [1]. In particular, BA10 is crucial for the ability to comply with an intended action in the future, i.e. a prospective memory (PM) task, like, for instance, to remember to stop by the bakery store to purchase some bread. Some authors suggest a functional dissociation between the medial (BA10med) and the lateral (BA10lat) portions of BA10 [2]. These observations rely upon functional neuroimaging studies that consistently detected an increase of the activity of BA10lat and a decrease of the activity of BA10med during prospective memory tasks. At present, it is not clear whether or not the functional dissociation between the two portions of BA10 can be interpreted within the theoretical framework of the gateway hypothesis [3,4]. According with this hypothesis, BA10 would bias attention toward external stimuli, stimulus-oriented though (SO), and internal representations, stimulus-independent though (SI). In particular, it has been suggested that during PM tasks the BA10lat mediates the SI attending whereas the BA10med the SO.

The aim of present study is to investigate the functional dissociation between BA10lat and BA10med by means of a functional imaging protocol. In particular, we manipulated the SI and the SO attending of PM cues assuming to modulate respectively brain activity within BA10lat and BA10med. For this reason we included in this study a functional localizer for the two portions of BA10 [4,5].

## 2. Methods

Sixteen (9 females) right-handed healthy young volunteers (aged 18–44 years) gave their written informed

ISSN 0953-4180/13/$27.50 © 2013 – IOS Press and the authors. All rights reserved

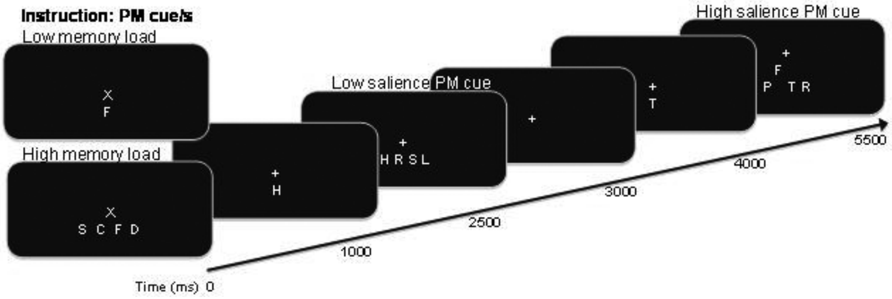

Fig. 1. Schematic representation of the experimental paradigm.

consent to participate to this study. The task [6] consisted in a continuous ongoing (ONG) task of letter search (Fig. 1). After the central presentation of a $1^\circ \times 1^\circ$ visual angle target letter (1 sec), a horizontal string of four letter was presented (1.5 sec) and the participant was asked to indicate whether the target was in the two right vs. left positions by pressing two different keys. At the beginning of each block of trials ($n = 40$) participants received the PM instruction asking to stop the ONG task and press a third key every time a particular PM cue letter was presented in one of the strings of the ONG task (in 25% of trials). During the task we varied orthogonally three factors in a $2 \times 2 \times 2$ design: the task (within blocks) with PM trials where the PM cue occurred and ONG trials where it did not occur; the saliency of the PM cue/ONG target (within blocks) that was high when they were presented not aligned respect to the other letters (HS) and low when they were aligned (LS); the memory load of the PM task (between blocks): high when cue letters presented were four (HM) and low when the cue was only one letter (LM). The functional localizer consisted in small blocks (10 trials) of ONG task alone or with the PM task, without any manipulation of saliency or memory load. Images were acquired with a T2*-weighted gradient-echo, echo-planar imaging (EPI) sequence on a 3T Siemens Allegra scanner (Siemens Medical Systems, Erlangen, Germany). Functional MR images were acquired using blood-oxygenation-level-dependent (BOLD) imaging covering the entire cortex (repetition time = 2.08 s, time echo = 30 ms, in-plane resolution = $3 \times 3$ mm, flip angle = $70^\circ$, slice thickness = 2.5 mm, inter-slice distance = 1.25 mm). The statistical analysis and image preprocessing was performed in SPM8 (Wellcome Department of Cognitive Neurology). After standard preprocessing, data were analyzed with the general linear model (GLM) for event-related designs. We modeled all the 8 condition of interest of the $2 \times 2 \times 2$ design (task $\times$ saliency $\times$ load) to perform a within-subjects analysis of variance, ANOVA. We averaged the bold signal (MarsBar 0.41, "MARseille Boite A Région d'Intéret" SPM toolbox) within two 10 mm radius spherical region of interests (ROI) centered on peaks of main activity within the BA10med and left BA10lat isolated with the functional localizer task.

## 3. Results

Within BA10lat emerged a significant interaction effect between task and memory load with maximal activity for PM trials in HM condition ($t = 2.22$, $p = 0.028$). Within BA10med emerged a significant main effect of saliency (HS > LS) ($t = 2.94$, $p = 0.004$) and a interaction between task and saliency, indicating a larger effect of saliency for PM trials, that approached significance ($t = 1.71$, $p = 0.09$).

## 4. Discussion

This study investigated the involvement of BA10 in a PM task, in particular the differential contribution of two portions of this area, lateral vs. medial, in two differential aspects of this complex ability. In the view of the gateway hypothesis, these two aspects are related to SO and SI thoughts [3,4]. In fact, during the processing of continuous stream of information (ongoing task), the competition between those two processes must be biased not only to maintain delayed intentions [7] but also transiently to perform the intention at the right moment (cue occurrence). In this study we orthogonally modulated the task (ONG vs. PM), SI processing (high vs. low memory load) and SO processing (high vs. low saliency). According with the gateway hypothesis, we found that the SI processing related to PM cues affected the BA10lat, whereas the SO processing affected the BA10mes for PM cues but, even if in a minor way, also for ONG targets. In line with the gateway hypothesis, for the first time, we demonstrated that two experimental manipulations, one affecting SO and the other SI, have different effects on the two portions of BA10 during execution of a PM task. From a theoretical point of view, this result suggests a "double functional dissociation" between lateral and medial portions of BA10.
